# Systematic Review and Meta-Analysis of Validation Studies on a Diabetes Case Definition from Health Administrative Records

**DOI:** 10.1371/journal.pone.0075256

**Published:** 2013-10-09

**Authors:** Aaron Leong, Kaberi Dasgupta, Sasha Bernatsky, Diane Lacaille, Antonio Avina-Zubieta, Elham Rahme

**Affiliations:** 1 Research Institute of the McGill University Health Centre, Montreal, Quebec, Canada; 2 Department of Medicine, McGill University, Montreal, Quebec, Canada; 3 Division of Rheumatology, Department of Medicine, University of British Columbia, British Columbia, Canada; Univserity of Tolima, Colombia

## Abstract

**Objectives:**

Health administrative data are frequently used for diabetes surveillance. We aimed to determine the sensitivity and specificity of a commonly-used diabetes case definition (two physician claims or one hospital discharge abstract record within a two-year period) and their potential effect on prevalence estimation.

**Methods:**

Following Preferred Reporting Items for Systematic Reviews and Meta-Analyses (PRISMA) guidelines, we searched Medline (from 1950) and Embase (from 1980) databases for validation studies through August 2012 (keywords: “diabetes mellitus”; “administrative databases”; “validation studies”). Reviewers abstracted data with standardized forms and assessed quality using Quality Assessment of Diagnostic Accuracy Studies (QUADAS) criteria. A generalized linear model approach to random-effects bivariate regression meta-analysis was used to pool sensitivity and specificity estimates. We applied correction factors derived from pooled sensitivity and specificity estimates to prevalence estimates from national surveillance reports and projected prevalence estimates over 10 years (to 2018).

**Results:**

The search strategy identified 1423 abstracts among which 11 studies were deemed relevant and reviewed; 6 of these reported sensitivity and specificity allowing pooling in a meta-analysis. Compared to surveys or medical records, sensitivity was 82.3% (95%CI 75.8, 87.4) and specificity was 97.9% (95%CI 96.5, 98.8). The diabetes case definition underestimated prevalence when it was ≤10.6% and overestimated prevalence otherwise.

**Conclusion:**

The diabetes case definition examined misses up to one fifth of diabetes cases and wrongly identifies diabetes in approximately 2% of the population. This may be sufficiently sensitive and specific for surveillance purposes, in particular monitoring prevalence trends. Applying correction factors to adjust prevalence estimates from this definition may be helpful to increase accuracy of estimates.

## Introduction

Diabetes is a leading cause of blindness, renal failure and cardiovascular disease [1]. The direct cost of diabetes and its complications put a substantial strain on healthcare system resources [2]–[4]. The rise in the prevalence of Type 2 diabetes has been largely driven by an ageing population, the obesity epidemic and a more sedentary lifestyle [5]. The prevalence of Type 1 diabetes is also on the rise [6], [7], although reasons for this increase are unclear. In order to adequately project needs and costs of diabetes management, it is crucial to know the actual prevalence of all diabetes and track changes over time.

Administrative databases have become a popular tool for diabetes research and disease surveillance, as they are less prone to recall bias, and potentially more cost efficient, than nationwide surveys [8]. Diabetes case identification algorithms can involve a combination of physician billing claims [9], hospitalization records [10], prescriptions data[10]–[12], and/or records of healthcare services utilization [13]. However, the validity of this method for prevalence estimation or diabetes research has not been definitively established.

There are several potential information gaps that can affect prevalence estimation from claims-based algorithms: first, regular patient use of the health care system is required for case identification; second, data coding for diabetes must be accurate and comprehensive; third, some physicians are not on a fee-for-service plan exclusively (i.e., they either receive a salary or are on a mixed remuneration plan) so visits to these physicians are not captured in some databases; and fourth, given that patients with diabetes commonly carry multiple comorbidities and are frequently managed by general practitioners [14], physicians may fill billing claims for conditions other than diabetes [15], [16].

The National Diabetes Surveillance System (NDSS) comprises regionally distributed diabetes surveillance systems across Canada and uses provincial administrative databases to identify diabetes cases and estimate population prevalence. According to the NDSS case definition, a diabetes case fulfils at least one of the following two criteria: two physician billing claims within a two-year period or one hospitalization with an ICD code for diabetes [17]. We note that administrative data and the ICD codes used do not distinguish between the two diseases.

Similar to other claims-based algorithms, the NDSS case definition may not be optimally sensitive for diabetes case identification. Thus we sought to (1) determine the overall NDSS case definition performance (sensitivity and specificity) through systematic review and meta-analysis, and (2) estimate diabetes prevalence adjusted for the performance of the NDSS case definition.

## Methods

### Search Strategy

The systematic review was conducted in accordance with the Preferred Reporting Items for Systematic Reviews and Meta-Analyses (PRSIMA) guidelines [18]. Two citation indices, Medline and Embase, were searched using an OVID platform. Keywords used included “administrative data”, “validation studies” and “diabetes mellitus” (Table S1 for search strings). The search strategy was limited to articles ever published through August 18, 2012 that were accessible via these search engines (i.e., from January 1, 1950 for Medline and from January 1, 1980 for Embase). The language of publication was not restricted. We also reviewed the bibliographies of relevant articles (i.e., citation tracking).

### Abstract Review and Abstract Exclusion Criteria

Each abstract was reviewed independently (AL and ER). We used the following inclusion criteria: (1) test measures were reported; (2) the validated case definition was similar to the NDSS algorithm; (3) the data sources were from administrative databases; (4) the study base was a representative sample of the general population and (5) the reference standard, via subject-specific record linkage, was adequate (e.g. self-report from population-based surveys, drug dispensation claims of anti-diabetic medication, laboratory data or primary care medical chart reviews). An example of an inadequate reference standard would be performing the validation test on a non-representative subsample of the study population. If the two investigators, AL and ER, disagreed, they attempted to reach consensus by discussion. A third investigator (KD) was consulted to serve as a tie-breaking adjudicator.

### Full-text Review, Quality Assessment and Data Extraction

Study quality was evaluated using Quality Assessment Tool for Diagnostic Accuracy Studies (QUADAS) criteria (Table S2) [19], as well as consideration of the following potential biases: (1) verification bias (was there a comparison with an independent reference standard with no knowledge of the index test results?), (2) spectrum bias (was there ample representation of patients commonly seen in clinical practice?), (3) review bias (were the index test results interpreted independently of the reference standard results?) and (4) incorporation bias (did the index test form part of the reference standard?). Study data were abstracted using standardized forms that recorded the following information: study population, data sources, administrative algorithms, validation method, reference standards, funding sources, sensitivity, specificity, positive predictive value (PPV), negative predictive value (NPV) and kappa statistic. If test measures or 95% confidence intervals were not reported in the original paper, estimates were calculated from data available. For example, we calculated the PPV from the sensitivity, specificity and prevalence when available using the following formula:
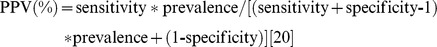



### Statistical Analysis

Using STATA version 11, we generated forest plots and summary receiver operating characteristic (SROC) curves to visually inspect for heterogeneity. Forest plots were arranged according to reference standard used, namely, self-report from surveys or medical chart review. Given that the sensitivity and specificity of each study are calculated from correlated binary outcomes, we judged that simple pooling using weighted average of the sensitivity and specificity independently was inadequate. Thus, we performed a DerSimonian & Laird random-effects bivariate regression analysis using a generalized linear mixed model approach that took heterogeneity and correlation between sensitivities and specificities into account [21], [22]. Pooled test accuracies were reported and hierarchical SROC curves were plotted (i.e., HSROC plots of sensitivity and specificity with 95% joint intervals in two-dimensional space). Confidence and prediction regions in the SROC space were constructed using the estimates from the bivariate normal distribution for the random-effects model.

Given the small number of studies, we were unable to perform meta-regression techniques or subgroup analyses to statistically describe the effect of study characteristics on the heterogeneity of test measures. Egger’s test and Begg’s funnel plots were not conducted because there was limited power to detect small-study effects of publication bias and these tests can be misleading in meta-analyses of diagnostic accuracy [23], [24].

### Additional Analyses

National surveillance reports of diabetes prevalence are not adjusted for the sensitivity and specificity of the diabetes case definition [25]. To demonstrate the effect of such adjustments on reported national surveillance results, we adjusted the yearly Canadian population prevalence of diabetes cases [25], using the pooled sensitivity and specificity derived from our study. Based on the law of total probability and Bayes Theorem, the correction formula generated to adjust prevalence was as follows [26]:
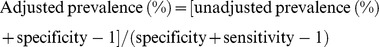



## Results

### Search Results

**Figure 1:**
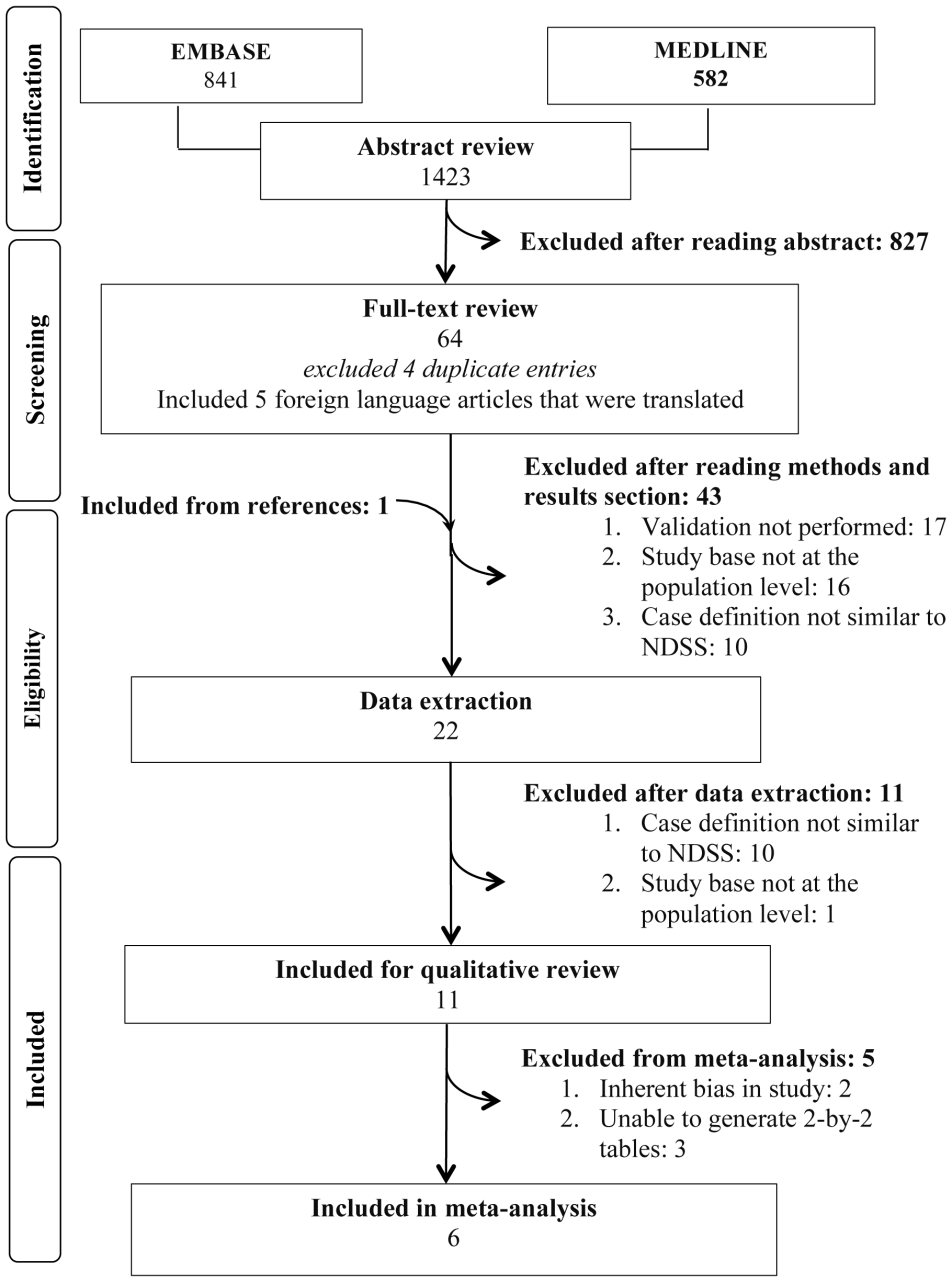
Flow diagram of selection strategy and article reviews. Flow diagram is in accordance with Preferred Reporting Items for Systematic Reviews and Meta-Analyses (PRISMA).

The search strategy identified 1423 abstracts. Among these, 65 were determined to be potentially relevant for full text review, of which five articles were published in a language other than English (one Danish, one Hebrew, two Italian and one Korean). The abstracts and method sections were translated by a native speaker of the language and determined not to be eligible for inclusion. A total of 43 studies were excluded for the following reasons: a validation was not performed; the study base was not representative of the general population; the validated case definition was too dissimilar from the NDSS case definition. Twenty two articles were considered for review and data extraction. A flow chart illustrating the selection process is shown in Figure 1.

At the time of full text review, five additional studies were excluded as they examined national registries rather than claims-based approaches[13], [27]–[30]. Five more studies were excluded because of important divergence in the case definition from that used in NDSS. Divergent algorithms excluded physician claims or only used one physician claim from the case definition, or included dispensation of anti-diabetic medication, biochemical information or physician reporting of patient diagnosis in the case definition. Another article was excluded because the study base was not representative of the general population. Ultimately, 11 studies met the eligibility criteria and were included in the systematic review. Included studies are displayed in Table 1.

**Table 1:** Test properties of the NDSS case definition.

Studypopulation	StudyYears	Author	Case definitionalgorithm	“Gold standard”/comparator	Age(years)	N	Sens (%)(95% CI)	Spec (%)(95% CI)	PPV (%)(95% CI)	NPV (%)(95% CI)	Kappa(95% CI)
Ontario,Canada	1992–1999	Hux [32] |	NDSS case definition from ODD	Prescription data in ODB	≥65	−	91.0	−	−	−	−
				Self-report in NPHS ◊	≥20	4691	85.0||(81.0, 89.0)	−	64.0||(59.0, 69.0)	−	−
				Medical records from GP offices	≥20	3317	86.1(82.0, 90.2)	97.1(96.5, 97.7)	79.8(76.0, 83.6)	98.1(97.6, 98.6)	0.80(0.77, 0.84)
	2006–2008	Harris [33] |	NDSS case definition from ODD	Medical records from EMR	Mean 42.5	19 442	84.3*(82.7, 86.3)	96.9*(96.4, 97.5)	74.8*(73.0, 76.6)	98.2*(98.0, 98.4)	0.77(0.75, 0.78)
	2000–2001	Shah ¢ Manuel [34]	NDSS case definition from ODD	Self-report in CCHS	≥18	1812	−	−	74.8*(72.8, 76.8)	−	−
Manitoba,Canada	1997–2002	Lix [36]	1) physician claims;2) Hospitalization;3) Prescription data from MHSIP	Self-report in CCHS	≥19	5589	−	−	−	−	0.74∫(0.73, 0.74)
	1989–1990	Robinson [9] |	1, 2 or 3 physician claim or1 hospitalization from MSHC@ ∫	Self-report in MHHP	18–74	2651	75.5* (69.2, 81.8)	97.8* (97.2, 98.4)	72.4* (66.1, 78.7)	98.1* (97.6, 98.6)	0.72 (0.67, 077)
Saskatchewan,Canada	1991–2001	Koleba [11]	NDSS case definition from SHB	Prescription claims data	≥20Mean 52.8	145 696	94.4(94.2, 94.6)^Δ^	−	−	−	−
Alberta andBritishColumbia,Canada	2001–2004	Southern [37]	1) 2 physician claim or 1 hospitalization@;2) 1 physician claim or 1 hospitalizationfrom AHS	Laboratory data from CLS¢	−	25 419	79.1(78.9, 79.4)	−	75.1(74.8, 75.3)	−	−
	2000–2002	Chen [38] |	1) 2 physician claims or 1 hospitalization@;2) 2 physician claims;3) 1 physician claim or 1 hospitalization;4) 1 physician claim	Medical record from GP offices	≥35Mean 53.2	3362	92.3(89.2, 95.5)	96.9(96.2, 97.5)	77.2(72.5, 81.7)	0.99(99.0, 99.6)	0.79(0.75, 0.83)
Minnesota,USA	2006	Solberg [31]	2 physician codes or1 hospitalization in1 year OR 1 anti-diabeticmedication from HEDIS	Prescription data, laboratory data and medical records	≥19	135 842	−	−	96.5(96.1, 96.9)	−	−
	1992–1994	O’Connor [39] |	2 outpatient physiciancodes from HMO	Self-report in HMO survey	Adults Mean 39.9	1976	76.1* (68.1, 84.1)	99.6* (99.3, 99.9)	92.2* (86.7, 97.7)	98.6* (98.1, 99.1)	0.83(0.78, 0.87)
	1992–1995	Hebert [40] |	14 algorithms that variedbetween 1 and 2 years, 1 or 2 Medicare claims@	Self-report in MCBS	≥65	7562	74.4(71.9, 76.9)	97.5(97.1, 97.9)	84.1(81.8, 86.4)	95.6(95.1, 96.1)	0.75(0.73, 0.78)

AHS: Alberta Health Services; CCHS: Canadian Community Health Survey; CLS: Calgary Laboratory Services; EMR: Electronic Medical Records; GP: General Practitioner; HEDIS: Health Plan Employer Data and Information Set; HMO: Health Maintenance Organization; Kappa: Kappa statistic; MCBS: Medicare Current Beneficiary Survey; MHSC: Manitoba Health Services Commission; MHHP: Manitoba Heart Health Project; MHSIP: Manitoba Health Services Insurance Plan; NDSS: National Diabetes Surveillance System; NPHS: National Population Health Survey; NPV: Negative predictive value; ODB: Ontario Drug Benefit; ODD: Ontario Diabetes Database; OHI: Ontario health Insurance Plan; Sens: Sensitivity; SHB: Saskatchewan health beneficiaries; Spec: Specificity; PPV: Positive predictive value.

| Included in meta-analysis.

◊ Test estimates and 95% confidence intervals which were not reported in the original paper were manually calculated from available raw data.

|| 95% confidence intervals were estimated based on a diabetes prevalence of 6.8% via the ODD and a sample size of 4691 from the NPHS cycle 3 1998/9.

* Test properties were recalculated to designate self-reported diabetes from survey as the reference standard.

The sample size of the ODB were not explicitly stated in the paper. Hence, the 95%CIs could not be calculated.

-Demographic data was not reported in the study.

∫ the authors validated one physician claim or one hospitalization which is similar but not identical to the NDSS criteria.

@test measures of the case algorithm closet to the NDSS criteria is displayed in the table.

¢ Test measures were calculated using the reference standard closest to current diabetes diagnosis criteria (i.e. CDA guidelines for diagnostic criteria of diabetes OR glycated hemoglobin ≥6.7%) was displayed in the table.

^△^The sensitivity was calculated by projecting the case-control design (n = 145 696) to the entire Saskatchewan population where 625 994 individuals would not have met the NDSS criteria of which 3443 would have been identified as having diabetes based in medication alone.

### Quality Assessment

The QUADAS scores (Table S3) ranged from 7 to 12 (median 11) out of a maximum of 14. The bias assessment identified two studies with potential deviations from our inclusion criteria. First, in the Solberg and colleagues’ study, investigators only reviewed the medical records of subjects who had tested positive in administrative data [31]. Therefore, the PPV could be reported in the paper but not the sensitivity or specificity without introducing verification bias. Second, the Koleba and colleagues study used prescription data both as part of the diabetes definition and also as the reference standard (incorporation bias) [11]. Regardless of quality assessment scores, all 11 studies are discussed in the systematic review.

### Qualitative Synthesis (Table 1)

All 11 studies were conducted in North America. Of these studies, eight were conducted in Canadian provinces (Three Ontario, two Manitoba, one Saskatchewan, one Alberta, and one Alberta/British Columbia) and three in Minnesota, USA.

Hux and colleagues [32] validated the Ontario Diabetes Database (ODD), a registry of diabetes cases identified by the NDSS algorithm (age ≥20 years; n = 528 280), through record linkage to three independent sources: first, a medication dispensation database from a public medication reimbursement plan for individuals ≥65 years of age; second, survey data from the National Population Health survey (NPHS; a stratified random sample that included query on diabetes); third, a random sample of medical charts (n = 3317) at physician offices in the community. Another Ontario study (mean age 42.5 years; n = 19 442) by Harris and colleagues [33] examined the concordance of the ODD with two other data sources: a provincial ICD-code based registry developed for the Baseline Diabetes Database Initiative (BDDI) by the Ontario Ministry of Health; and anti-hyperglycemic medication prescriptions, laboratory test results and physician-recorded lists of medical diagnosis (i.e., problem lists) in electronic medical records from primary care practices residing in rural and urban areas of southwestern Ontario as part of the Delivery Primary Health-care Information (DELPHI) project [33]. In a different Ontario study, Shah & Manuel [34] validated the ODD against self-report from the Canadian Community Health Survey (CCHS) (age ≥18 years; n = 1812), a cross-sectional survey of health determinants, health status and health care use in the Canadian population [35].

In Manitoba, Robinson and colleagues [9] examined one, two or three physician contacts, defined by physician service claims or hospital summaries from the Manitoba Health Services Commission (MHSC) database (age range 18–74 years; n = 2792). The reference standard was self-report from the Manitoba Heart Health Project (MHHP), a population-based cross-sectional health survey. Lix and colleagues [36] validated 152 case definition algorithms derived from physician claims, prescription data and hospitalization data on chronic diseases, including diabetes, from Manitoba Health Services Insurance Plan (MHSIP) administrative data with CCHS (age ≥19 years; n = 5589). Of note, the Manitoba drug benefit program (i.e. Pharmacare) covers all Manitobans and reimbursement is scaled according to taxable income and amount of prescription drug cost.

Koleba and colleagues [11] determined the case capture rate of the NDSS definition in Saskatchewan using drug dispensation records (mean age 52.8 years; n = 145 696). Approximately 90% of the Saskatchewan population are eligible for public prescription benefits.

In Calgary, Southern and colleagues [37] validated the NDSS case definition and a more liberal definition involving single physician claims on a defined cohort of diabetes cases diagnosed by laboratory criteria of elevated fasting or post-prandial blood glucose values, or glycated hemoglobin levels (all ages; n = 25 419).

Chen and colleagues [38] performed their validation study on both rural and urban populations of Alberta and British Columbia and compared algorithms that varied in number of physician claims against medical records. General practitioner clinics were randomly selected from urban and rural areas and medical charts were randomly selected from within each clinic’s patient registries (mean age 52.8 years; n = 3362).

The three Minnesota studies had different study designs to validate claims-based administrative algorithms similar to the NDSS algorithm. First, O’Connor and colleagues [39] validated computerized insurance databases of Health Maintenance Organization (HMO) members in the Upper Midwest with self-report from a telephone survey (adults; mean age 40 years; n = 3186). Discordant cases had their medical charts reviewed. Second, Hebert et al [40] used self-reported diabetes from the Medicare Current Beneficiary Survey (MCBS) to validate an administrative algorithm from claims data of Medicare beneficiaries. This study, however, was performed only on individuals ≥65 years of age who had comprehensive Medicare coverage. Thus, the specificity may be higher among these individuals because of more frequent physician encounters compared to a younger population. The claims data included those pertaining to home health agencies in addition to claims for hospitalizations and outpatient physician encounters. Third, Solberg and colleagues [31] reviewed medical records on a random sample of Medicare beneficiaries in Minnesota to verify the diabetes status of cases identified through NDSS-like case definitions from the Health Plan Employer Data and Information Set (HEDIS; age ≥19 years; n = 135 842).

### Test Performance of the NDSS Algorithm Against Self-report in Surveys (Table 1)

Hux and colleagues reported a sensitivity of 85.0% (95%CI 81.0, 89.0%) and a PPV of 64.0% (95%CI 59.0, 69.0%) when the ODD was compared to self-report from NPHS (cycle three health component, 1998/1999; n = 4691). The 95% confidence intervals were estimated based on a diabetes prevalence of 6.8% in the ODD [32]. Shah and Manual yielded a higher PPV of 74.8% (95%CI 72.8, 76.8%) when the ODD was compared to self-reported diabetes from CCHS (reference standard); as the study cohort was established entirely with diabetes cases identified from the ODD, the sensitivity of the ODD could not be calculated [34]. In Manitoba, Robinson and colleagues reported a more modest sensitivity of 75.5% (95%CI 69.2, 81.8%) coupled with a high specificity [98.1% (95%CI 97.6, 98.6%)] [9]. These test measures were similar to those reported by the two American studies by O’Connor and colleagues and Hebert and colleagues [sensitivities: 76.1% (95%CI 86.1, 84.1%) and 74.4% (95%CI 71.9, 76.9%), respectively; specificities: 99.6% (95%CI 99.3, 99.9%) and 97.5% (95%CI 97.1, 97.9%), respectively] [39], [40].

### Test Performance of the NDSS Algorithm Against Medical Records/Laboratory Data/Prescription Dispensation Data (Table 1)

Chen and colleagues in Alberta/British Columbia demonstrated high sensitivity [92.3% (95%CI 89.2, 95.5%)] and specificity [96.9% (95%CI 96.2, 97.5%)] of the NDSS algorithm against medical records [38]. Hux and colleagues reported a slightly lower sensitivity [86.1% (95%CI 82.0, 90.2%)] but comparable specificity [97.1% (95%CI 76.5, 97.7%)] using ODD data [32]. A similar sensitivity [84.3% (95%CI 82.7, 86.3%)] and specificity [96.9% (95%CI 96.4, 97.5%)] of the NDSS algorithm against electronic medical records were found by Harris and colleagues [33]. Southern and colleagues yielded a slightly lower sensitivity of 79.1% (78.9, 79.4%) when administrative data from Alberta Health Services were compared to laboratory data [37].

In general, high sensitivities were reported for the NDSS case definition against prescription data, such as the ODB (sensitivity; 91.0%, sample size not available to calculate the 95%CI) by Hux and colleagues for individuals ≥65 years of age [32]. A similar sensitivity was reported by Koleba and colleagues among adults ≥20 years of age who were Saskatchewan Health Beneficiaries [sensitivity; 94.4% (95%CI 94.2, 94.6%)] when results were projected to the entire Saskatchewan population [11].

The NDSS case definition had generally good concordance with medical records (kappa 0.77–0.80) and self-reported diabetes from surveys (kappa 0.72–0.83). In general, higher concordance between case ascertainment techniques (e.g. diabetes cases from administrative case definitions and self-report from surveys) was reported by American studies compared to Canadian studies.

### Meta-analysis

**Figure 2:**
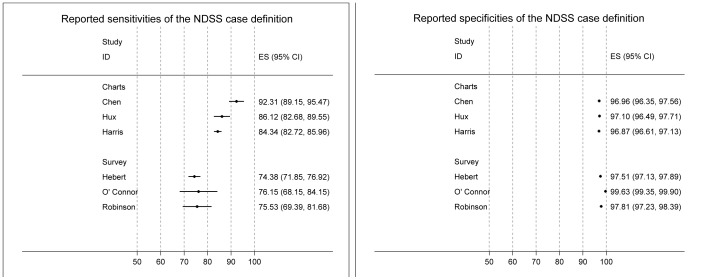
Forest plots of sensitivities and specificities of the NDSS case definition reported by included validation studies. ES (95%CI): Summary estimate (95% confidence interval); Charts: Reference standard by medical chart review; Survey: Reference standard by patient self-report from population-based survey.

We were able to populate four-cell values of diagnostic two-by-two tables from available raw data of 6 studies [9], [32], [33], [38]–[40]. The reported sensitivities ranged from 74.4% to 92.3% (median 85.2%) and specificities ranged from 96.9% to 99.6% (median 97.3%, Figure 2). Studies validated by surveys (n = 3) had lower sensitivities (74.4% to 76.2%) than those validated by medical records (n = 3; 84.3% to 92.3%). The area under the curve (AUC) of the symmetric SROC was 97.7% (95%CI 97.1, 98.3%) and asymmetric SROC was 96.8% (95%CI 92.1, 100.0%) for all 6 studies (Figure 2).

**Figure 3:**
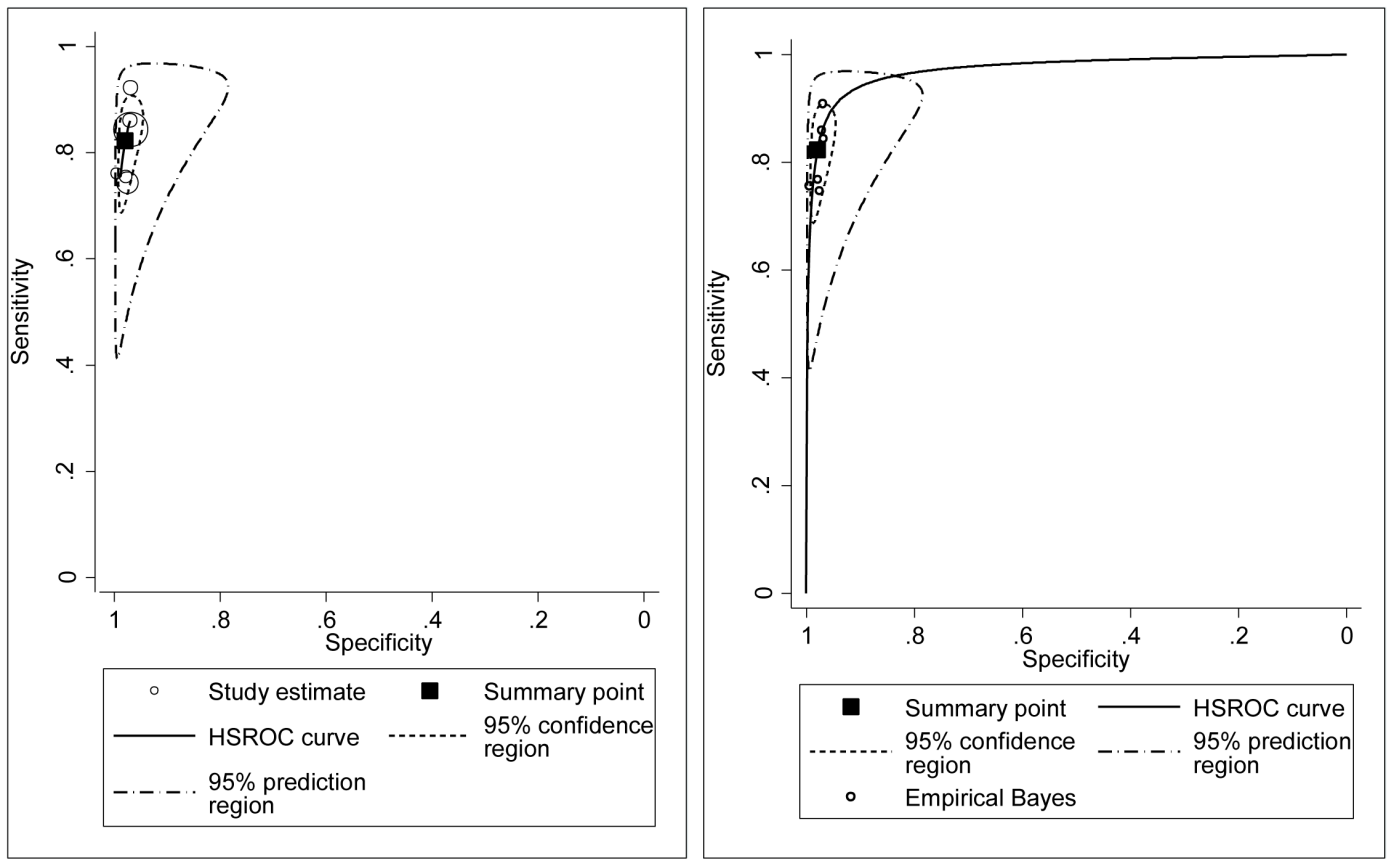
Random-effects bivariate regression analysis of the pooled test accuracies from 6 studies. The Hierarchical Summary Receiver Operator Characteristics (HSROC) curve displays the 95% confidence interval of the summary operating point and the 95% prediction region, which is the confidence region for a forecast of the true sensitivity and specificity in a future study. The shape of the prediction region is generated based on the assumption of a bivariate normal distribution for the random effects model. The Empirical Bayes estimate gives the best estimate of the true sensitivity and specificity of each study and these estimates will be shrunk towards the summary point compared with the study-specific estimates. The stronger the shrinkage, the greater the precision of the test estimate. The random-effects bivariate regression analysis could not be done for the subgroups stratified by validation method because the small number of studies.

By random-effects bivariate regression analysis, the overall pooled sensitivity was 82.3% (95%CI 75.8, 87.4%) and specificity was 97.9% (95%CI 96.5, 98.8%, Figure 3). The 95% prediction region, which is the confidence region for a forecast of the true sensitivity and specificity in a future study, ranged more widely from under 50% to over 90% for the predicted sensitivity and from approximately 80% to almost 100% for the predicted specificity. A multi-level hierarchical model and random-effects bivariate regression model for subgroups by validation method could not be performed because of the small number of studies (Figure 3).

### Additional Analyses

NDSS reports prevalence estimates of physician-diagnosed diabetes as the proportion of cases identified via the NDSS case definition in the population. This study demonstrated that the NDSS case definition is not gold standard and misclassifies ∼ 20% of diabetes cases and ∼ 2% of non-cases. From the Canadian 2009 NDSS report [25], the yearly population prevalence rates of NDSS-identified diabetes cases among adults aged ≥20 years between fiscal years 2002/3 and 2006/7 were adjusted by applying the following correction factors based on the pooled test accuracies (sensitivity and specificity) of the NDSS case definition:

Adjusted prevalence (%) = [reported unadjusted prevalence (%) - 2.1%]/80.2%.

**Figure 4:**
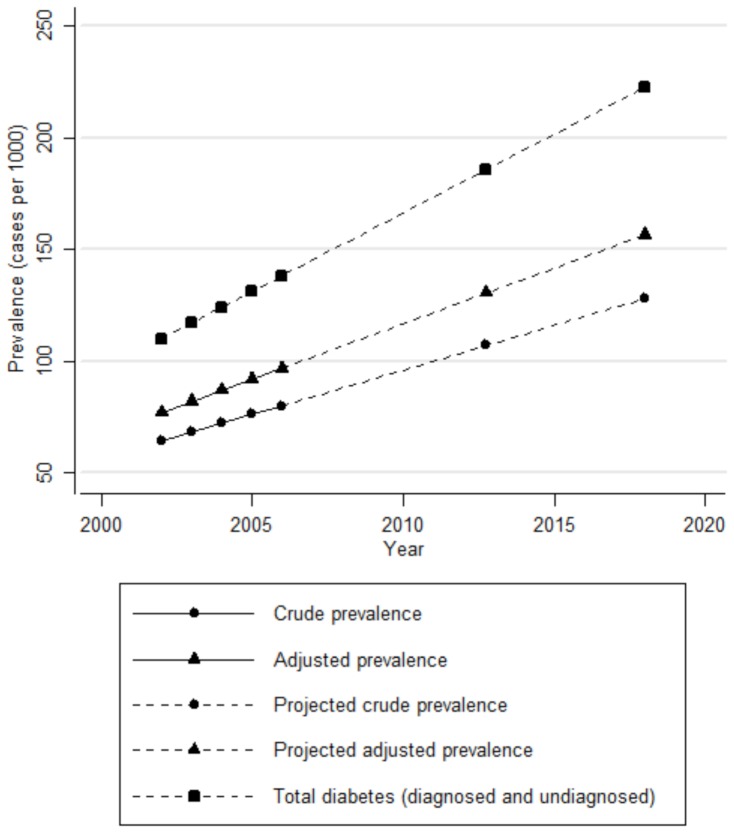
Crude and adjusted prevalence of diabetes in Canada. Crude prevalence: prevalence of diabetes in Canada for fiscal years 2002/3 through 2006/7 obtained from the NDSS 2009 report [25]; Adjusted prevalence: prevalence after applying correction factors [(Prevalence(%) - 2.1)/0.802)]; The margins of error for all adjusted prevalence and crude prevalence estimates were ∼0.01% (n∼25 000 000). Projected crude prevalence: future prevalence assuming an increase of 0.4% per year; Projected adjusted prevalence: future prevalence after applying correction factors; Total diabetes: Estimated prevalence of physician-diagnosed and undiagnosed diabetes assuming 1/3 of total diabetes is undiagnosed. The crossover point of the crude and adjusted prevalence lines is ∼10.6% around year 2013.

Figure 4 shows adjusted and unadjusted prevalence estimates plotted against time from fiscal year 2002/3 to 2006/7, respectively. These prevalence estimates were then projected over 10 years (to year 2018). The 95% margin of error for all adjusted and unadjusted prevalence estimates were ≤0.01% (population size, n ∼ 25 000 000).

The impact of prevalence adjustment depended on the magnitude of diabetes prevalence. Unadjusted prevalence estimates were biased upwards by ∼ 1% during the 5-year period (2002/3: unadjusted prevalence was 6.4% and adjusted prevalence was 5.3%; 2006/7: unadjusted prevalence was 8.0% and adjusted prevalence was 7.3%). However, the NDSS case definition underestimated the increase in prevalence over time as reflected by the steeper slope for adjusted prevalence against time (∼ 0.4% per year) compared to unadjusted prevalence against time (∼ 0.5% per year). Both unadjusted and adjusted prevalence equaled 10.6%, around year 2013. This crossover point occurred when the number of false positives equaled to the number of false negatives. After year 2013, unadjusted prevalence estimates appear biased downwards.

As the PPVs were not consistently provided in the included studies, they were not pooled. Instead, we estimated the PPV based on the pooled NDSS sensitivity and specificity presented herein, using the following formula:

PPV(%) = sensitivity*prevalence/[(sensitivity+specificity-1)*prevalence+(1-specificity)] [20]; using the pooled test measures reported herein, PPV(%) = [82.3*prevalence(%)]/[0.802*prevalence(%)+2.1]. Assuming diabetes prevalence is between 5% and 10%, the PPV falls between 67.3% and 81.3%.

## Discussion

Our meta-analysis demonstrates that a commonly-used administrative database definition for diabetes (2 physician outpatient billings and/or one hospitalization with a diabetes record on the discharge abstract summary within a two-year period) has a pooled sensitivity of 82.3% (95%CI 75.8, 87.4) and specificity of 97.9% (95%CI 96.5, 98.8%), based on the findings of 6 studies with complete data available. While this definition appears to miss approximately one fifth of diabetes cases and wrongly classifies 2.1% of non-cases in the population as diabetes cases, it is likely sufficiently sensitive for monitoring prevalence trends in the general population if its accuracy remains reasonably stable over time [41]. In such situations, this administrative database definition can be particularly useful for tracking prevalence changes over time.

In a previous examination of administrative database definitions for diabetes, Saydah and colleagues [42] performed a literature review of validation studies on a variety of diabetes administrative database definitions, gold standards and patient populations, from highly restrictive (e.g. only patients who underwent percutaneous coronary interventions) to nationally representative. The authors included 16 validation studies and reported that diabetes administrative database definitions varied from moderately to very sensitive [46.0% to 97.0% (median 81.5%)] but were uniformly very specific [95.0% to 100.0% (median 99.0%)]. The authors did not perform a meta-analysis in that study. Our study focused specifically on the evaluation of the NDSS definition and found its sensitivity to range from 74.4% to 94.4% with a median of 81.7%; this median is similar to the median sensitivity of all diabetes administrative database definitions examined by Saydah and colleagues.

It has been suggested that the sensitivity of a claims-based administrative algorithm could potentially be improved by incorporating information from medication dispensation data, without compromising specificity [11]. However, some regions have restricted public medication insurance coverage; therefore prevalence estimation from prescription data may not always be representative of the general population. While medication dispensation information may improve the sensitivity among those reimbursed by the public healthcare system, not all people are covered by the government drug plan. This can bias results non-differentially through improving the estimate in the group with coverage but not in the group of individuals without coverage.

The high specificity of administrative database case definitions cannot be under appreciated as it contributes to a low false positive rate and high PPV, thus reducing the potential for overestimating prevalence. A PPV above 70% has been deemed sufficient for surveillance of other health outcomes (e.g., cerebrovascular accidents, congestive heart failure and venothromboembolism) [43]. We demonstrated that the PPV of the NDSS case definition is generally higher than 70% assuming true diabetes prevalence is >5%. If diabetes prevalence is <5%, over a third of diabetes cases may in fact be falsely identified as diabetes cases. Conversely, a prevalence >10% reduces the false positive rate which renders the NDSS case definition more efficient. In this situation, however, the NDSS case definition could underestimate prevalence if the number of false negative cases exceeds that of false positive cases. Above all, the choice of administrative database diabetes case definition depends on the underlying prevalence of the disease and the goals of the surveillance system that might warrant maximizing the sensitivity at the expense of some specificity and PPV.

Sudden or marked changes in diabetes prevalence should prompt a re-validation of the test accuracy of the case definition [41]. Thereafter, yearly change in diabetes prevalence can be adjusted and better quantified through applying correction factors derived from the test accuracies of the case definition. From fiscal year 2002/3 to 2006/7, the NDSS case definition underestimated the rise in diabetes prevalence in Canada by approximately 0.4% (78,625 diabetes cases) over the 5-year period (Table S4). The importance of applying correction factors grows over time as the bias appears to rise with increasing diabetes prevalence. While administrative databases are unable to distinguish between type 1 and type 2 diabetes, the majority of cases among adults have type 2 diabetes; thus fluctuations in overall diabetes prevalence likely reflect changes in type 2 diabetes prevalence.

Administrative case definitions and medical chart reviews both generally capture advanced physician-diagnosed diabetes cases and frequent users of health services. This potentially explains the higher sensitivity and concordance when administrative case definitions were compared with medical chart reviews than with surveys. While estimating the prevalence of advanced cases is important for health economics and manpower distribution, infrequent users of health services and individuals with diabetes that have not been brought to medical attention are likely to have downstream diabetes-related complications and attendant healthcare utilization. Indeed, none of the included validation studies accounted for undiagnosed diabetes. It has been previously estimated that approximately one third of diabetes cases remain undiagnosed [44]–[46]. Accounting for undiagnosed diabetes not only increases the prevalence of diabetes considerably but also steepens the increase in diabetes prevalence over time as shown in Figure 4.

### Reference Standards

There are potential limitations for all reference standards used to validate administrative definitions for diabetes. The accuracy of primary care charts reviews depends largely on physician charting, availability of records, and the accurate interpretation of medical data during the review process. Medical chart reviews miss cases in the general population if diabetes screening is not routinely performed on every patient in the primary care setting. Poor participation by physicians also introduces bias, as physicians who agree to participate may have a keener interest in diabetes care, more thorough diabetes evaluations and follow-ups for patients in their practice and/or clearer medical charting.

Information bias could be introduced in surveys through patients’ poor recall, social desirability bias, poor understanding of survey questions, or incomplete knowledge of their diagnoses. The extremes of age are more likely to report having diabetes [47], [48] and the effect of sex could influence reporting in either direction[47]–[49]. Both lower education [50] and poorly-controlled diabetes have been found to be associated with underreporting [51]. Surveys can also suffer from participation biases as asymptomatic individuals with low diabetes risk may be less willing to participate whereas certain patients with advance diabetes may be too unwell to participate.

We acknowledge that the correction factors proposed herein were based on the premise that medical chart reviews and population-based surveys had perfect sensitivity and specificity. In the absence of a “gold standard” for validating administrative algorithms [52], Bayesian statistical approaches, that incorporate the uncertainties of non-gold standard case ascertainment techniques, could be undertaken to estimate the true population prevalence [53]. Alternatively, a thorough assessment of sensitivity measurements obtained via different reference standards can be performed to corroborate prevalence estimates and surveillance results from administrative data [41], [54].

### Strengths and Limitations

Our systematic review was comprehensive as it had a broad search strategy that bore no language or time restriction. Foreign language articles were partially translated by colleagues who were native speakers of these languages. Study selection was performed by two independent reviewers and discrepancies were adjudicated by a third reviewer. It was likely that only a small number of relevant articles were missed by our search strategy which was generic and based on the intercept of only a few keywords. The bibliographies of included studies were also perused. While only two major electronic databases (Medline and Embase) were examined, it was felt that other search engines, such as Cochrane Central Register of Controlled Trials (CENTRAL), would unlikely yield any study of interest given that validation studies are not designed as randomized controlled trials. The inclusion of unpublished studies might arguably reduce publication bias but expose the review to lower quality studies that potentially lack rigorous statistical techniques of published studies [55]. All 11 included studies captured patient information at the population level with clear case definitions, were validated by reference standards encompassing a broad spectrum of patients and had QUADAS scores over 10. These studies were funded by large research agencies and academic centres (Table S5) with no reported disclosures from the private sector or special interest groups.

The heterogeneity observed in the meta-analysis likely arose from different reference standards used. Other potential sources of heterogeneity are differences in socio-demographic characteristics, geographical location, year of study, health insurance arrangements, physician remuneration schemes, prescription subsidies and healthcare utilization, practices and access. Therefore, a random-effects bivariate regression model that accounted for heterogeneity and correlation between sensitivity and specificity was used to pool the test measure estimates. Heterogeneity could also result from misclassification due to unmeasured confounders, such as human error in physician claims and hospitalizations coding. However, this was unlikely as the administrative databases studied have been previously validated and used widely for research studies and surveillance efforts.

### Generalizability

As all included studies were conducted in North America, we assumed that study bases were similar enough to make direct comparisons between studies. We found generally good concordance (kappa statistic >0.7) between the cases identified through the administrative data versus medical records and the administrative data versus population-based surveys across studies, suggesting that public administrative data are a viable substitute for these other case ascertainment methods. Given that administrative data conveniently encompasses the entire population in identifying diabetes cases, it is particularly efficient for national surveillance. Indeed, maintaining a nationwide diabetes registry is expensive for a chronic disease as prevalent as diabetes.

However, while study bases were nested in the general population, the selected study samples were not always random and, thus, may not necessarily be representative of the total population. Mild variations in the statistical agreement between administrative data and medical records/surveys might be explained by differences in the constitution of the study bases. Higher concordance was reported between the administrative database case definition and medical records/surveys in the American studies which were conducted on well-defined populations (e.g., within a HMO). Conversely, slightly lower concordance was found in the Canadian studies that studied agreement between the NDSS case definition and self-report from surveys targeting the entire population via stratified random sampling.

Extrapolation of the pooled test measures of the NDSS algorithm to other jurisdictions, with different healthcare systems, administrative databases, physician remuneration arrangements and patient populations, demands caution. This also highlights the need for jurisdictions to periodically evaluate the test accuracies of administrative algorithms on new populations. As the stability of sensitivity measurements is essential to monitor disease trends over time, validation studies should be repeatedly performed at different time point within the same population.

In sum, claims-based algorithms are widely used across North America and play a vital role in Canadian diabetes surveillance strategies. Thus, establishing the criterion validity of the NDSS case definition is critical for healthcare professionals and public health researchers. We have shown that the NDSS case definition has an acceptable sensitivity and a reasonably high specificity for diabetes surveillance. By applying correction factors to reported diabetes prevalence from Canadian surveillance reports, we demonstrated that the NDSS case definition overestimates prevalence when it is ≤10.6% and does the converse when prevalence is >10.6%; hence, correction factors can be applied to make proper quantifications of yearly prevalence. Even with the use of correction factors to account for the NDSS test accuracies, the administrative database algorithm probably misses new or mild diabetes and is unable to identify undiagnosed diabetes cases. It does, however, capture advanced physician-diagnosed diabetes cases and frequent users of healthcare services. Estimating the population prevalence of these diabetes cases is important for health services, health economics, and budget and manpower allocation.

## Supporting Information

Table S1 **MEDLINE and EMBASE Search strategies.**
(DOCX)

Table S2 **The QUADAS tool.** The QUADAS tool was extracted from table 2 of Whiting, P., et al., *The development of QUADAS: a tool for the quality assessment of studies of diagnostic accuracy included in systematic reviews.* BMC Med Res Methodol, 2003. 3: p. 25. [19].
(DOCX)

Table S3 **Quality assessment by QUADAS.** Questions were selected from QUADAS to constitute the “*Bias Assessment*”. QUADAS questions are displayed in Table S2.
(DOCX)

Table S4 **Adjusted and unadjusted prevalence of diabetes in Canada.** Diabetes prevalence rates from fiscal year 2002/3 to 2006/7 of individuals aged ≥20 years from the NDSS 2009 report were adjusted using the following correction formula: [prevalence (%) –2.1]/0.802. Based on the sample size of ∼25,000,000 individuals in Canada, the margin of error was ∼0.01% for all adjusted and prevalence estimates. The adjusted cost of diabetes per year is consistently lower than the estimated cost calculated from diabetes cases identified by the NDSS. However, the increase in adjusted diabetes prevalence over the 5-year time span is greater by 0.4% than the crude prevalence. This amounts to an additional 78625 diabetes cases that would not have been accounted for without the application of correction factors.
(DOCX)

Table S5 **Funding sources of included validation studies.**
(DOCX)

Checklist S1 **PRISMA Checklist.**
(DOCX)
