# Supplementary material for: Systematic Review and Meta-Analysis of Validation Studies on a Diabetes Case Definition from Health Administrative Records
Source: PLoS One. 2013 Oct 9;8(10):e75256. doi: 10.1371/journal.pone.0075256 (PMC3793995; doi:10.1371/journal.pone.0075256)
Supplement: Table S1 — MEDLINE and EMBASE Search strategies. (DOCX) [file pone.0075256.s001.docx]

| **Table S1: MEDLINE and EMBASE Search strategies**  MEDLINE  Database: Ovid MEDLINE(R) 1950 to Present with Daily Update, Ovid MEDLINE(R) In-Process & Other Non-Indexed Citations <November 15, 2010>  Database: Ovid MEDLINE(R) 1950 to Present with Daily Update, Ovid MEDLINE(R) In-Process & Other Non-Indexed Citations <November 15, 2010>  Search Strategy:  --------------------------------------------------------------------------------  1 administrative data.ti,ab. (2391)  2 administrative database:.ti,ab. (1329)  3 Databases, Factual/ (30843)  4 factual database/ (30843)  5 Databases as Topic/ (7263)  6 database/ (0)  7 Medical Record Linkage/ (2778)  8 administrative databank:.ti,ab. (2)  9 factual database:.ti,ab. (17)  10 factual databank:.ti,ab. (2)  11 factual data.ti,ab. (53)  12 exp medical records/ (69234)  13 exp medical record/ (69234)  14 exp medical records systems, computerized/ (18265)  15 (medical record or health record or medical records or health records).ti,ab. (45693)  16 medical transcription:.ti,ab. (73)  17 exp Registries/ (42227)  18 registry/ (39948)  19 (registry or registries).ti,ab. (41377)  20 (utilization data: or utilisation data: or claims data: or managed care data: or physician billing data: or hospitalization data: or linked data:).ti,ab. (5478)  21 (administrative healthcare data: or administrative health care data: or administrative health data: or administrative health data:).ti,ab. (142)  22 (medical records based index or claims based index).ti,ab. (5)  23 (register and (link or links or linked or linkage or linking)).ti,ab. (1850)  24 or/1-23 [ADMINISTRATIVE DATA (BROAD)] (206366)  25 Validation Studies/ (47964)  26 validation study/ (0)  27 Validation Studies as Topic/ (588)  28 Validation Studies.pt. (47964)  29 (validat: or validity).ti,ab. (236536)  30 or/25-29 [VALIDATION STUDIES] (257352)  --------------------------------------------------------------------------------------  98 exp Diabetes Mellitus/ (261426)  99 diabet:.ti,ab. (319880)  100 98 or 99 [DIABETES MELLITUS (BROAD)] (366136)  101 24 and 30 and 100 [ADMINISTRATIVE DATA (BROAD) + VALIDATION STUDIES + DIABETES MELLITUS (BROAD)] (438)  102 diabetes mellitus.ti,ab. (97761)  103 98 or 102 [DIABETES MELLITUS (NARROW)] (287652)  104 24 and 30 and 103 [ADMINISTRATIVE DATA (BROAD) + VALIDATION STUDIES + DIABETES MELLITUS (NARROW)] (296) SAVED EN 2143-2438 (277 UNIQUE)  105 31 and 103 [ADMINISTRATIVE DATA (NARROWEST) + DIABETES MELLITUS (NARROW)] (214)  106 104 or 105 (476)  107 105 not 104 (180) SAVED EN 2439-2618 (165 UNIQUE)  108 (comorbidity index or comorbidity indexes or comorbidity indices).ti,ab. [COMORBIDITY INDEXES] (995)  109 24 and 30 and 108 [ADMINISTRATIVE DATA (BROAD) + VALIDATION STUDIES + COMORBIDITY INDEXES] (44) SAVED EN 2619-2662 (42 UNIQUE)  110 31 and 108 [ADMINISTRATIVE DATA (NARROWEST) + COMORBIDITY INDEXES] (63)  111 109 or 110 (88)  112 110 not 109 (44) SAVED EN 2663-2706 (41 UNIQUE)  MEDLINE / EMBASE  Database: EMBASE <1980 to 2010 Week 45>, Ovid MEDLINE(R) 1950 to Present with Daily Update, Ovid MEDLINE(R) In-Process & Other Non-Indexed Citations <November 15, 2010>  Search Strategy:  --------------------------------------------------------------------------------  1 administrative data.ti,ab. (4927)  2 administrative database:.ti,ab. (2833)  3 Databases, Factual/ (41452)  4 factual database/ (41452)  5 Databases as Topic/ (79396)  6 database/ (72133)  7 Medical Record Linkage/ (92752)  8 administrative databank:.ti,ab. (4)  9 factual database:.ti,ab. (41)  10 factual databank:.ti,ab. (6)  11 factual data.ti,ab. (110)  12 exp medical records/ (159208)  13 exp medical record/ (159208)  14 exp medical records systems, computerized/ (108239)  15 (medical record or health record or medical records or health records).ti,ab. (96352)  16 medical transcription:.ti,ab. (150)  17 exp Registries/ (73671)  18 registry/ (71392)  19 (registry or registries).ti,ab. (88310)  20 (utilization data: or utilisation data: or claims data: or managed care data: or physician billing data: or hospitalization data: or linked data:).ti,ab. (11879)  21 (administrative healthcare data: or administrative health care data: or administrative health data: or administrative health data:).ti,ab. (296)  22 (medical records based index or claims based index).ti,ab. (10)  23 (register and (link or links or linked or linkage or linking)).ti,ab. (3848)  24 or/1-23 [ADMINISTRATIVE DATA (BROAD)] (466927)  25 Validation Studies/ (72226)  26 validation study/ (24262)  27 Validation Studies as Topic/ (24850)  28 Validation Studies.pt. (47964)  29 (validat: or validity).ti,ab. (504774)  30 or/25-29 [VALIDATION STUDIES] (533582)  ----------------------------------------------------------------------------------  98 exp Diabetes Mellitus/ (665833)  99 diabet:.ti,ab. (696125)  100 98 or 99 [DIABETES MELLITUS (BROAD)] (839087)  101 24 and 30 and 100 [ADMINISTRATIVE DATA (BROAD) + VALIDATION STUDIES + DIABETES MELLITUS (BROAD)] (1027)  102 diabetes mellitus.ti,ab. (211612)  103 98 or 102 [DIABETES MELLITUS (NARROW)] (706730)  104 24 and 30 and 103 [ADMINISTRATIVE DATA (BROAD) + VALIDATION STUDIES + DIABETES MELLITUS (NARROW)] (810)  105 31 and 103 [ADMINISTRATIVE DATA (NARROWEST) + DIABETES MELLITUS (NARROW)] (554)  106 104 or 105 (1276)  107 105 not 104 (466)  108 (comorbidity index or comorbidity indexes or comorbidity indices).ti,ab. [COMORBIDITY INDEXES] (2258)  109 24 and 30 and 108 [ADMINISTRATIVE DATA (BROAD) + VALIDATION STUDIES + COMORBIDITY INDEXES] (89)  110 31 and 108 [ADMINISTRATIVE DATA (NARROWEST) + COMORBIDITY INDEXES] (128)  111 109 or 110 (180)  112 110 not 109 (91)  ----------------------------------------------------------------------------------  129 remove duplicates from 104 (592)  130 from 129 keep 1-316 [EMBASE: ADMINISTRATIVE DATA (BROAD) + VALIDATION STUDIES + DIABETES MELLITUS] (316) SAVED EN 3634-3949 (314 UNIQUE)  131 remove duplicates from 107 (291)  132 from 131 keep 1-127 [EMBASE: ADMINISTRATIVE DATA (NARROWEST) + DIABETES MELLITUS] (127) SAVED EN 3950-4076 (127 UNIQUE)  ----------------------------------------------------------------------------------- |
| --- |
