# Supplementary material for: Systematic Review and Meta-Analysis of Validation Studies on a Diabetes Case Definition from Health Administrative Records
Source: PLoS One. 2013 Oct 9;8(10):e75256. doi: 10.1371/journal.pone.0075256 (PMC3793995; doi:10.1371/journal.pone.0075256)
Supplement: Table S2 — The QUADAS tool. The QUADAS tool was extracted from table 2 of Whiting, P., et al., The development of QUADAS: a tool for the quality assessment of studies of diagnostic accuracy included in systematic reviews. BMC Med Res Methodol, 2003. 3: p. 25. [19]. (DOCX) [file pone.0075256.s002.docx]

**Table S2: The QUADAS tool**

The QUADAS tool was extracted from table 2 of Whiting, P., et al., *The development of QUADAS: a tool for the quality assessment of studies of diagnostic accuracy included in systematic reviews.* BMC Med Res Methodol, 2003. 3: p. 25.
