# Supplementary material for: Systematic Review and Meta-Analysis of Validation Studies on a Diabetes Case Definition from Health Administrative Records
Source: PLoS One. 2013 Oct 9;8(10):e75256. doi: 10.1371/journal.pone.0075256 (PMC3793995; doi:10.1371/journal.pone.0075256)
Supplement: Table S3 — Quality assessment by QUADAS. Questions were selected from QUADAS to constitute the “Bias Assessment”. QUADAS questions are displayed in Table S2. (DOCX) [file pone.0075256.s003.docx]

**Table S3: Quality assessment by QUADAS**

|  | **Studies included in the qualitative review / meta-analysis** | | | | | | **Other studies in the qualitative review** | | | | |
| --- | --- | --- | --- | --- | --- | --- | --- | --- | --- | --- | --- |
| **QUADAS Question no.** | **Chen** [[36](#_ENREF_36)] | **Harris**  [[31](#_ENREF_31)] | **Hebert** [[38](#_ENREF_38)] | **Hux**  [[30](#_ENREF_30)] | **O’Connor** [[37](#_ENREF_37)] | **Robinson** [[7](#_ENREF_7)] | **Koleba**  [[9](#_ENREF_9)] | **Lix**  [[34](#_ENREF_34)] | **Shah**  [[32](#_ENREF_32)] | **Solberg** [[29](#_ENREF_29)] | **Southern** [[35](#_ENREF_35)] |
| **1*** | Y | Y | Y | Y | Y | Y | Y | Y | Y | Y | Y |
| **2** | Y | Y | Y | Y | Y | Y | Y | Y | Y | Y | Y |
| **3** | Y | Y | Y | Y | Y | Y | N | Y | Y | Y | Y |
| **4** | Y | Y | Y | Y | Y | Y | Y | Y | Y | Y | Y |
| **5** | Y | Y | Y | Y | Y | Y | N | Y | Y | N | Y |
| **6*** | Y | Y | Y | Y | Y | Y | N | Y | Y | N | N |
| **7** | Y | Y | Y | Y | Y | Y | Y | Y | Y | N | Y |
| **8** | Y | Y | Y | Y | Y | Y | Y | Y | Y | Y | Y |
| **9** | Y | Y | Y | Y | Y | Y | Y | Y | Y | Y | Y |
| **10*** | Y | Y | Y | Y | Y | Y | Y | Y | Y | Y | Y |
| **11*** | Y | Y | Y | Y | Y | Y | N | Y | Y | N | Y |
| **12** | Unclear | Unclear | Unclear | Unclear | Unclear | Unclear | Unclear | Unclear | Unclear | Unclear | Unclear |
| **13** | N | N | Y | N | Y | Y | N | Unclear | N | N | Y |
| **14** | Unclear | Unclear | Unclear | Unclear | Unclear | Unclear | Unclear | Unclear | Unclear | Unclear | Unclear |
| **Score**  **(max 14)** | 11 | 11 | 12 | 11 | 12 | 12 | 7 | 11 | 11 | 7 | 12 |
| **Bias assessment (max 5)** | 5 | 5 | 5 | 5 | 5 | 5 | 3 | 5 | 5 | 2 | 5 |

*Questions were selected from QUADAS to constitute the “*Bias Assessment*”. QUADAS questions are displayed in Table S2.
