# Supplementary material for: Systematic Review and Meta-Analysis of Validation Studies on a Diabetes Case Definition from Health Administrative Records
Source: PLoS One. 2013 Oct 9;8(10):e75256. doi: 10.1371/journal.pone.0075256 (PMC3793995; doi:10.1371/journal.pone.0075256)
Supplement: Table S4 — Adjusted and unadjusted prevalence of diabetes in Canada. Diabetes prevalence rates from fiscal year 2002/3 to 2006/7 of individuals aged ≥20 years from the NDSS 2009 report were adjusted using the following correction formula: [prevalence (%) –2.1]/0.802. Based on the sample size of ∼25,000,000 individuals in Canada, the margin of error was ∼0.01% for all adjusted and prevalence estimates. The adjusted cost of diabetes per year is consistently lower than the estimated cost calculated from diabetes cases identified by the NDSS. However, the increase in adjusted diabetes prevalence over the 5-year time span is greater by 0.4% than the crude prevalence. This amounts to an additional 78625 diabetes cases that would not have been accounted for without the application of correction factors. (DOCX) [file pone.0075256.s004.docx]

**Table S4: Adjusted and unadjusted prevalence of diabetes in Canada**

| **Fiscal Year** | **Unadjusted Prevalence (%)** | **Adjusted Prevalence (%)** | **Difference in Prevalence (%)** | **Cases, n** | **Adjusted cases, n** | **Difference in cases, n** | **Population, n** |
| --- | --- | --- | --- | --- | --- | --- | --- |
| 2002/3 | 6.4 | 5.4 | -1.0 | 1,540,001 | 1,293,996 | -246,005 | 24,134,536 |
| 2003/4 | 6.8 | 5.9 | -0.9 | 1,657,282 | 1,437,778 | -219,504 | 24,534,007 |
| 2004/5 | 7.2 | 6.4 | -0.8 | 1,781,879 | 1,584,653 | -197,226 | 24,919,442 |
| 2005/6 | 7.6 | 6.9 | -0.7 | 1,916,172 | 1,736,332 | -179,840 | 25,318,884 |
| 2006/7 | 8.0 | 7.4 | -0.6 | 2,061,995 | 1,894,615 | -167,380 | 25,753,921 |
| Change in 5 years | 1.6 | 2.0 | 0.4 | 521,994 | 600,619 | 78,625 | 1,619,385 |

Diabetes prevalence rates from fiscal year 2002/3 to 2006/7 of individuals aged ≥20 years from the NDSS 2009 report were adjusted using the following correction formula: [prevalence (%) – 2.1]/0.802. Based on the sample size of ~25,000,000 individuals in Canada, the margin of error was ~0.01% for all adjusted and prevalence estimates. The adjusted cost of diabetes per year is consistently lower than the estimated cost calculated from diabetes cases identified by the NDSS. However, the increase in adjusted diabetes prevalence over the 5-year time span is greater by 0.4% than the crude prevalence. This amounts to an additional 78625 diabetes cases that would not have been accounted for without the application of correction factors.
