# Supplementary material for: Systematic Review and Meta-Analysis of Validation Studies on a Diabetes Case Definition from Health Administrative Records
Source: PLoS One. 2013 Oct 9;8(10):e75256. doi: 10.1371/journal.pone.0075256 (PMC3793995; doi:10.1371/journal.pone.0075256)
Supplement: Table S5 — Funding sources of included validation studies. (DOCX) [file pone.0075256.s005.docx]

**Table S5: Funding sources of included validation studies**

| **Title** | **population** | **Author** | **Funding sources** |
| --- | --- | --- | --- |
| Investigating concordance in diabetes diagnosis between primary care charts (electronic medical records) and health administrative data: a retrospective cohort study | Ontario, Canada | Harris | Institute for Clinical Evaluative Sciences  (ICES) |
| Diabetes in Ontario: determination of prevalence and incidence using a validated administrative data algorithm. |  | Hux | Medical Research Council of Canada |
| Self-reported diabetes is associated with self-management behaviour: a cohort study |  | Shah & Manuel | Canadian Diabetes Association; CIHR |
| Population-based data sources for chronic disease surveillance. | Manitoba, Canada | Lix | CIHR |
| Estimating the burden of disease. Comparing administrative data and self-reports |  | Robinson | Heart and stroke foundation |
| Prescription drug data and the national diabetes surveillance system case definition. | Saskatchewan, Canada | Koleba | CIHR |
| Validating ICD coding algorithms for diabetes mellitus from administrative data | Alberta and British Columbia, Canada | Chen | CIHR |
| Validity of administrative data claim-based methods for identifying individuals with diabetes at a population level |  | Southern | Canadian Diabetes Association |
| Identifying persons with diabetes using Medicare claims data | Minnesota, USA | Hebert | Association of Schools of Public Health/ Communicable disease Centre; Prevention Cooperative Agreement |
| Identifying diabetes mellitus or heart disease among health maintenance organization members: Sensitivity, specificity, predictive value, and cost of survey and database methods |  | O’Connor | Health Partners Research Foundation |
| Are claims data accurate enough to identify patients for performance measures or quality improvement? The case of diabetes, heart disease, and depression. |  | Solberg | Robert Wood Johnson Foundation |
